# Sleep fragmentation increases blood pressure and is associated with alterations in the gut microbiome and fecal metabolome in rats

**DOI:** 10.1152/physiolgenomics.00039.2020

**Published:** 2020-06-22

**Authors:** Katherine A. Maki, Larisa A. Burke, Michael W. Calik, Miki Watanabe-Chailland, Dagmar Sweeney, Lindsey E. Romick-Rosendale, Stefan J. Green, Anne M. Fink

**Affiliations:** ^1^Department of Biobehavioral Health Science, College of Nursing, University of Illinois at Chicago, Chicago, Illinois; ^2^Nursing Department, Nursing Research and Translational Science, National Institutes of Health, Clinical Center, Bethesda, Maryland; ^3^Office of Research Facilitation, College of Nursing, University of Illinois at Chicago, Chicago, Illinois; ^4^NMR-Based Metabolomics Core, Cincinnati Children's Hospital Medical Center, Cincinnati, Ohio; ^5^Genome Research Core, Research Resources Center, University of Illinois at Chicago, Chicago, Illinois

**Keywords:** cardiovascular diseases, circadian rhythm, metabolic syndrome, metabolite, microbiota, sleep disorders

## Abstract

The gut microbiota, via the production of metabolites entering the circulation, plays a role in blood pressure regulation. Blood pressure is also affected by the characteristics of sleep. To date, no studies have examined relationships among the gut microbiota/metabolites, blood pressure, and sleep. We hypothesized that fragmented sleep is associated with elevated mean arterial pressure, an altered and dysbiotic gut microbial community, and changes in fecal metabolites. In our model system, rats were randomized to 8 h of sleep fragmentation during the rest phase (light phase) or were undisturbed (controls) for 28 consecutive days. Rats underwent sleep and blood pressure recordings, and fecal samples were analyzed during: baseline (*days −4* to *−1*), early sleep fragmentation (*days 0–3*), midsleep fragmentation (*days 6–13*), late sleep fragmentation (*days 20–27*), and recovery/rest (*days 28–34*). Less sleep per hour during the sleep fragmentation period was associated with increased mean arterial pressure. Analyses of gut microbial communities and metabolites revealed that putative short chain fatty acid-producing bacteria were differentially abundant between control and intervention animals during mid-/late sleep fragmentation and recovery. Midsleep fragmentation was also characterized by lower alpha diversity, lower Firmicutes:Bacteroidetes ratio, and higher Proteobacteria in intervention rats. Elevated putative succinate-producing bacteria and acetate-producing bacteria were associated with lower and higher mean arterial pressure, respectively, and untargeted metabolomics analysis demonstrates that certain fecal metabolites are significantly correlated with blood pressure. These data reveal associations between sleep fragmentation, mean arterial pressure, and the gut microbiome/fecal metabolome and provide insight to links between disrupted sleep and cardiovascular pathology.

## INTRODUCTION

The metabolites of bacterial carbohydrate fermentation in the colon affect blood pressure (31, 68, 78), suggesting an important role for the gut microbiota in cardiovascular health and disease (22, 39). Short chain fatty acids (SCFAs), which include butyrate, propionate, and acetate, are produced by fermentative intestinal microbes, and these metabolites enter the circulation via the portal vein (15). Prior studies have shown that metabolites produced by the gut microbiota, such as SCFAs and succinate, can increase blood pressure by stimulation of renin release and activation of the renin-angiotensin system (40, 63, 67). Furthermore, elevated fecal levels of SCFAs were correlated with increases in blood pressure in humans and rodents (22, 43). Although there is a strong body of research linking elevated blood pressure with compositional changes in the bacterial communities of the gut microbiota (2, 28, 44, 50, 58, 78), the mechanisms by which gut microbiota metabolites affect blood pressure has not been thoroughly examined. Therefore, advancing understanding of fecal metabolome and blood pressure relationships may provide insight to the metabolic consequences and systemic effects that result in changes in the bacterial communities of the gut microbiota.

Sleep fragmentation (SF) and circadian rhythm disruption are linked to deleterious cardiovascular changes in humans and rodents. Previous research showed chronic SF during the light phase was associated with increased sympathetic activity, structural elastic vascular changes, and infiltration of macrophages and foam cells in the aorta of rodents (18, 48). Interestingly, sleep deprivation also caused endothelial changes in sympathectomized rats, suggesting that cardiovascular changes are not purely a result of sympathetic overactivation (69). In human populations, SF was associated with subclinical markers and cardiovascular disorders such as arterial stiffness, coronary artery calcium, hypertension, metabolic syndrome, and peripheral vascular disease (7, 26, 46, 47). Although these findings demonstrate that hypertension may be a consequence of the vascular endothelial changes resulting from SF, blood pressure responses after termination of SF and the associated mechanisms have not been studied.

The timing and activity of vital physiologic processes are regulated by central (suprachiasmatic nucleus) and peripheral oscillations. Peripheral oscillators, found in organs such as the liver, heart, and muscle, and the gut microbiota depend on input cues of food intake, sleep, and activity to determine circadian rhythms (33, 62, 72). SF interrupts the homeostasis of these daily oscillations, potentially leading to metabolic syndrome, a condition that may be the result of alterations in the gut microbiota (41, 45, 64). Furthermore, proinflammatory gut microbiota alterations that occur with many disorders, such as an increase in Proteobacteria, indicate a possible mechanism for the cardiovascular changes found with disordered sleep (29, 71). This may represent a reversible mechanism for the increased cardiovascular disease risk that has been linked to SF and sleep disorders (7, 47, 76). Due to the complex and bidirectional effects between SF and the microbiome and SF and cardiovascular physiology, the relationship between elevated blood pressure, a hallmark of metabolic syndrome, and the gut microbiota has not yet been studied in the setting of SF. A thorough understanding of the temporal responses of the gut microbiota community and blood pressure to SF is needed to provide a framework for future research.

There is a critical knowledge deficit in the understanding of mechanisms linking unhealthy sleep patterns with increased blood pressure and elevated cardiovascular disease risk. Furthermore, it is unknown whether blood pressure changes are sustained after SF is discontinued. The purpose of the present study was to examine the effects of SF, a highly common feature of human sleep pathology, on changes in the gut microbiota, metabolite production, and blood pressure. A specific objective of the study was to determine whether 8 h of daily SF, lasting 28 days, affected the gut microbiota community structure and function in rats. In addition, we sought to identify biological features in the gut microbiota (microbial taxa and metabolites) that were associated with changes in blood pressure.

## MATERIALS AND METHODS

### 

#### Animals.

Procedures conformed to the American Physiological Society’s *Guiding Principles for the Care and Use of Vertebrate Animals* (4) and were approved by the University of Illinois at Chicago Animal Care and Use Committee. Adult male Wistar-Kyoto rats (*n* = 15, 8–10 wk of age) were obtained from Charles River Laboratories (Kingston, NY). Rats were randomized to be controls (*n* = 7) or to undergo SF (*n* = 8). For control rats, the sample size decreased from seven (*days −4* to *19*) to six rats (*days 20* to *34*), and for SF rats, the sample size decreased from eight (*days −4* to *19*) to six rats (*days 20* to *34*). The two control rats and three SF rats were removed from the study at *day 20* because of issues with the transmitter implantation sites; data from these rats were used in analysis. Throughout the study, rats were housed in a temperature- and light-controlled room [66–76°F; lights on at Zeitgeber time (ZT) 0, lights off at ZT 12].

#### Surgical procedures.

Rats were anesthetized with 2–3% isoflurane and given 1 mg/kg buprenorphine SR LAB subcutaneously for implantation of two telemetry transmitters [models HDS11 and F40-EET, Data Sciences International (DSI), Minneapolis, MN]. To measure blood pressure and heart rate, a catheter was advanced into the abdominal aorta to a position caudal to the renal bifurcation; the HD-S11 telemetry battery was sutured beneath the abdominal muscle. During the same surgery, two stainless steel screw electrodes were placed in the skull (2.0 mm rostral and 1.0 mm medial/lateral from the bregma) to measure the cortical electroencephalogram (EEG), and bipolar electrodes were inserted into the nuchal muscles to record the electromyogram (EMG). The F40-EET battery was placed subcutaneously via a flank incision.

#### SF chambers.

After surgery, rats recovered for 1 wk before acclimation to specialized chambers for fragmenting sleep. The SF chambers provided a living space of 24 cm by 19 cm by 19 cm and ad libitum access to food and water (Lafayette Neuroscience, Lafayette, IN). Both groups were fed normal chow (Teklad Irradiated LM-485 Rat Diet 7912; Envigo, Madison, WI) and housed on corn cob bedding (Envigo) in the SF chambers. For 3 days before data collection rats explored the SF chambers so that the introduction of this environment did not affect sleep or cardiovascular parameters. Similar to other studies (27, 34), the SF chambers were used to impair rats’ ability to obtain sustained periods of deep sleep. When activated, the SF chamber fragmented sleep using a timer-controlled metal bar that swept horizontally along the bottom of the SF chamber. Considering that rats are nocturnal animals with an increased propensity for sleep during the light phase, we programmed the SF chambers’ sweeping bar movement to occur between ZT 1 and ZT 9 when the recording room lights were on. The movement of the bar from one side to the other side of the cage lasted 7 s (moving 4,080 times in the 8 h period) to briefly awaken rats when they fell asleep.

#### Data collection.

**Fig. 1.**
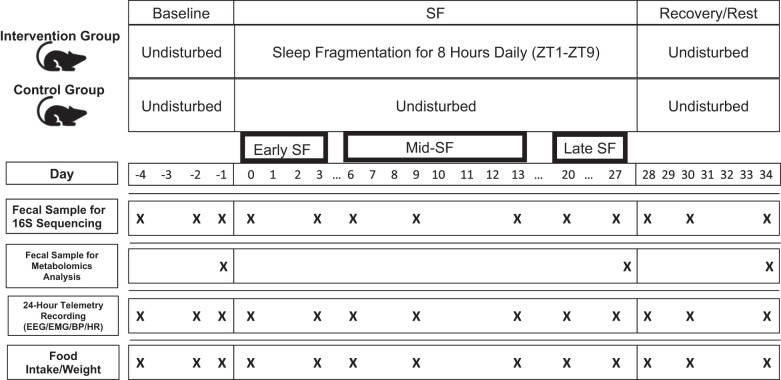
Study timeline and variables. Animals were randomized to intervention (8 h of sleep fragmentation daily for 28 days) or control conditions. Fecal samples were collected for 16S rRNA gene amplicon sequencing analysis 3 times during baseline, 7 times during the intervention period, and 3 times during recovery/rest. Additional fecal samples were collected on the last day of baseline, the last day of intervention, and the last day of recovery for metabolomics analysis. We analyzed 24 h telemetry recordings and food intake/weight on all days that fecal samples were collected. Abbreviations: 16S, 16S rRNA gene amplicon sequencing; BP, blood pressure; EEG, encephalogram; EMG, electromyogram; HR, heart rate; SF, sleep fragmentation; ZT, Zeitgeber time.

The days of the study, and the variables recorded, are shown in Fig. 1. The EEG, EMG, blood pressure, and heart rate signals from the telemetry transmitters were acquired continuously during the study, which lasted a total of 38 days (RPC-3 receivers and Ponemah software version 5.3, DSI). Signals were amplified, filtered, and digitized with a sampling rate of 500 Hz.

As illustrated in Fig. 1, baseline recordings were obtained for 3 days to measure undisturbed sleep and mean arterial pressure (MAP; *days −4*, *−2*, and *−1*). The rats were randomized to have fragmented sleep (SF, *n* = *8*) or to be undisturbed (*n* = *7*) for the next 27 days; the undisturbed rats served as controls. Control rats were also housed in the SF chambers, but the sweeping bar was never activated. After 28 days, both groups underwent a 7-day recovery/rest period during which all rats could sleep ad libitum. Body weight and food consumption were quantified every 24 h at ZT 4. Similar to others (75), feeding efficiency was calculated by dividing the daily weight gain from baseline (grams; baseline average of *days −4, −2,* and *−1*) by daily food intake (grams).

**Fig. 2.**
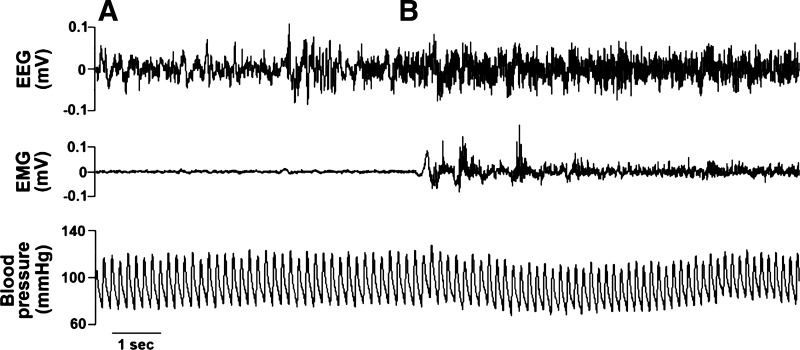
Representative tracings of the electroencephalogram (EEG), electromyogram (EMG), and blood pressure during sleep and wakefulness. Physiologic changes as a rat awakens from sleep. *A*: sleep, which is defined by relatively low frequency/high amplitude EEG activity and low EMG tone. *B***:** a transition from sleep to wakefulness; when the rat is awake, EEG waves become desynchronized, increase in frequency, and decrease in amplitude. EMG activity increases when the rat becomes physically active. More variability in blood pressure occurs when the rat is awake and active.

The EEG/EMG signals were scored offline using NeuroScore (version 3.0, DSI, Minneapolis, MN). Each 10 s epoch of the EEG and EMG was scored using an automated algorithm and classified as wake or sleep based on EEG cortical, EEG theta, EMG neck, and activity inputs (14). Wakefulness was defined as high-frequency, low-amplitude EEG with high EMG tone and/or activity levels above 0.1 counts; nonrapid eye movement (NREM) sleep was defined as increased spindle and delta ratio > 1, decreased EMG tone, and activity levels below 0.1 counts; and REM sleep was defined as high-frequency, low-amplitude EEG with a theta to delta ratio of 3, low EMG tone, and activity levels below 0.1 counts (Fig. 2). All scoring was visually confirmed by manual review for accuracy. NREM and REM sleep time were combined for the “total sleep time” variable, and total sleep (min/h) was evaluated during the bar on period (ZT 1–ZT 9).

#### Microbiome characterization.

Fecal samples were collected for microbiome characterization through microbial 16S ribosomal RNA (rRNA) gene amplicon sequencing and metabolomics analysis (Fig. 1). Freshly voided fecal pellets were collected at a consistent time (ZT 10) to minimize circadian fluctuations in microbiome community structure (51). Genomic DNA was extracted from fecal pellets using a DNeasy PowerFecal Kit (Qiagen, Valencia, CA), following the manufacturer’s protocol. Genomic DNA was prepared for high-throughput next-generation sequencing using a two-stage protocol (37), amplifying the V4 variable region of the 16S rRNA gene using primers 515F (modified) and 806R (modified) (74). Barcoded amplicons from each sample were pooled and sequenced on an Illumina MiniSeq instrument. DNA extraction, library preparation, and sequencing were performed by the Genome Research Core at the University of Illinois at Chicago.

Basic processing of the raw data was performed by the University of Illinois at Chicago Research Informatics Core. Raw sequence data were processed using the software package QIIME2 (16). Chimeras were identified and removed, and rarefaction was performed to exclude samples with insufficient reads per sample counts. Closed-reference taxonomical classification was performed using USEARCH (30) and the silva_132_16S.97 reference database (65). Abundance data were binned and summarized in biological observation matrices (BIOMs) for taxonomic levels from phylum to species.

#### Metabolomics sample preparation and analysis.

Additional fecal samples (intervention *days −1*, *27*, and *34*; Fig. 1) were collected for metabolomics analysis and sent to the nuclear magnetic resonance (NMR)-based Metabolomics Core at Cincinnati Children’s Hospital for processing. Stool samples were lyophilized for 3 days and homogenized into fine power by a cryogenic ball-mill homogenizer (Cryomill, Retsch). Each sample was weighed (100 ± 3 mg) into 2 mL tubes containing 2.8 mm ceramic beads (VWR) before processing. On the day of processing, 1.0 mL of cold PBS was added to each tube and the samples were homogenized for 30 s at 5,000 rpm using Minilys (Bertin Technologies). Samples were centrifuged at 5,000 *g_n_* at 4°C for 10 min. The supernatants were further centrifuged at 10,000 *g_n_* at 4°C for 15 min, twice each, in new 2 mL tubes. Supernatants were filtered at 12,000 *g_n_* for 90 min at 4°C using prewashed 3 kDa spin filters (NANOSEP 3K, Pall Life Sciences). The NMR buffer containing 100 mM phosphate buffer in D_2_O, pH 7.3, and 1.0 mM TMSP (3-trimethylsilyl 2,2,3,3-d_4_ propionate) was added to 300 μL of fecal filtrate. The final sample volume was 600 μL and final TMSP concentration in each sample was 0.5 mM.

Experiments were conducted using 550 μL samples in 103.5 × 5 mm NMR tubes (Bruker Analytik, Rheinstetten, Germany). One-dimensional ^1^H-NOESY NMR spectra were acquired on a Bruker Avance II 600 MHz spectrometer with 5 mm, BBO Prodigy probe. All data were collected at a calibrated temperature of 298°K using the noesygppr1d pulse sequence in the Bruker pulse sequence library. Experiments were run with four dummy scans and 1k acquisition scans with an acquisition time of 2.4 s and a relaxation delay of 3.0 s. The NOESY mixing time was 6 ms. The spectral width was 12 ppm, and 64K real data points were collected. All free induction decays were subjected to an exponential line-broadening of 0.3 Hz. Upon Fourier transformation, each spectrum was manually phased, baseline corrected, and referenced to the internal standard TMSP at 0.0 ppm using Topspin 3.6 software (Bruker). Two-dimensional data, ^1^H-^1^H total correlation spectroscopy (TOCSY) and ^1^H-^13^C heteronuclear single quantum coherence (HSQC), were collected for metabolite assignment on representative samples. For metabolomics analysis, metabolites were assigned by comparing the chemical shifts with reference spectra found in databases, such as the Human Metabolome Database (77) and Chenomx NMR Suite profiling software (Chenomx version 8.1). A total of 45 metabolites were assigned and quantified using Chenomx software based on the internal standard (Supplemental Table S1; https://doi.org/10.6084/m9.figshare.12149148). Prior to statistical analysis, the data were normalized by dry sample weights (μmol/mg).

#### Statistical analyses.

Statistical analyses were performed in GraphPad Prism version 8.21 for OS X, IBM SPSS Statistics 22 (New York, NY), and Stata Statistical Software: release 14. College Station, TX: StataCorp. Normality of the data was tested by a Ryan-Joiner test and transformed where the R value of the raw data was above the critical R at 0.05, indicating normally distributed data. Univariate comparisons were made between variables with two groups with *t* tests. When analyzing variables over several timepoints, we used mixed model analysis, using group (control, SF) as a fixed effect and animal as a repeated measure, followed by post hoc multiple comparison tests with Sidak’s correction for individual comparisons when appropriate. Repeated covariance structure was chosen according to the best-fit Schwarz’s Bayesian information criterion. Differences were indicated as statistically significant if *P* ≤ 0.05. Results are presented as means ± SE.

Microbiome BIOM files were used for downstream ordinations and statistical tests, implemented within the software packages QIIME2 (16), MicrobiomeAnalyst (24), and the “vegan” and “phyloseq” packages within the R programming environment (v. 3.5.1; R Foundation for Statistical Computing, Vienna, Austria) (25, 57, 66). Composition of individual gut microbiota samples were evaluated by the Shannon alpha diversity index to measure the richness and evenness of individual bacterial communities. To evaluate the compositional difference between SF and control fecal samples, beta diversity was calculated using a Bray-Curtis dissimilarity matrix, and relationships between intervention and control samples were tested using analysis of similarities (ANOSIM) (20). Differential abundance of specific taxa between intervention days was calculated using the parametric DESeq2 test on raw species level counts (54). To determine putative SCFA- and succinate-producing bacteria groups, bacterial genera were stratified according to their primary fermentation products as acetate, butyrate, propionate, or succinate (5, 10, 11), and fold-change in relative abundance of putative SCFA- and succinate-producing bacteria at the genus level across intervention periods was quantified (Supplemental Table S2; https://doi.org/10.6084/m9.figshare.12149202).

Mixed-effects regression models were run to test for the changes in gut microbiota alpha diversity, and the relative abundance of specific microbial taxa of interest across time for the SF and control rats. Microbial taxa were selected either by a priori hypothesis (i.e., SCFA-producing taxa), were identified in untargeted DESeq2 analysis of microbial community analyses, or were putative producers of metabolites of interest. Results for these models are presented as the average positive or negative change across time. For the relative abundance of Firmicutes bacteria, the trend of time varied depending on exposure to the treatment condition. Therefore, separate slopes for the different time periods were calculated via the use of a time spline, an additional time variable added to the regression equation that is coded as zero before the slope change and increase for each unit of time afterwards. To test for the overall effect of metabolites and microbiome measures on cardiovascular outcomes, mixed models were conducted in which each metabolite and microbiome measure was regressed on blood pressure outcomes (MAP), while controlling for time and treatment group. For these models, the regression coefficient for each metabolite and microbiome measure is reported which represents the change in cardiovascular outcome (e.g., increase/decrease in MAP) per a 1 unit increase in the microbiome measure or per a 0.1 unit increase in the metabolite measure.

#### Data availability.

The raw sequencing data acquired in this study have been deposited to the National Center for Biotechnology Information Sequence Read Archive under the accession code PRJNA625755. Cardiovascular, sleep, food intake, weight and metabolite data are available in Supplemental Table S1; https://doi.org/10.6084/m9.figshare.12149148. Taxonomic classification from phylum to species and abundance data (raw counts) of fecal microbiome samples available in Supplemental Table S3; https://doi.org/10.6084/m9.figshare.12149235.

## RESULTS

We analyzed 24 h MAP and fecal samples from control and SF rats on 13 separate days to characterize the temporal effects of SF on microbial community structure, composition, and diversity. In addition to cultivation-independent molecular analysis of microbial communities, direct measurement of gut microbiota metabolites was performed to identify treatment effects on metabolite profiles.

### 

#### SF impaired sleep, and SF rats had higher 24 h MAP than controls.

**Fig. 3.**
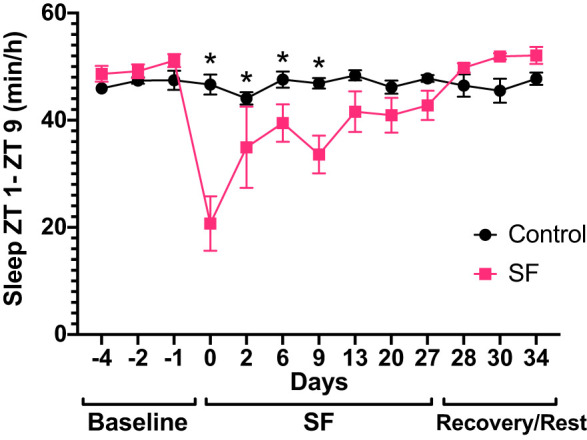
Sleep during the 8 h sleep fragmentation (SF) period in Control and SF rats. Total sleep/hour (h) during the 8 h (SF chamber bar on) SF period [Zeitgeber time (ZT) 1 to ZT 9]. Data (means ± SE) were analyzed using mixed model analysis with repeated/fixed measures (time × SF/control groups) followed by post hoc multiple comparison tests with Sidak’s correction. *P < 0.05. *Days −4* to *13* include 7 control and 8 SF rats; *Days 20* to *34* include 6 control and 6 SF rats.

Sleep during the 8 h segment of the light phase (ZT 1–9) is illustrated in Fig. 3, showing comparisons between control and SF rats. After the baseline period, during which control and SF rats had similar amounts of sleep, the ability of SF rats to sleep was affected by activating the SF chamber (group × time *F*_12, 93.41_ = 7.1, *P* < 0.001). Post hoc analysis demonstrated that SF rats had significantly less sleep than controls at *days 0* through *9*. We restricted the sleep comparisons to the 8 h SF time period because the SF rats resumed normal sleeping activities each day once the SF chamber turned off at ZT 9. When the SF bar was turned off (ZT 9–ZT 23 and ZT 0), sleep was not statistically different between SF and control rats, indicating that SF rats did not recover from SF by increasing sleep during the remaining 16 h of each day (data not shown).

**Fig. 4.**
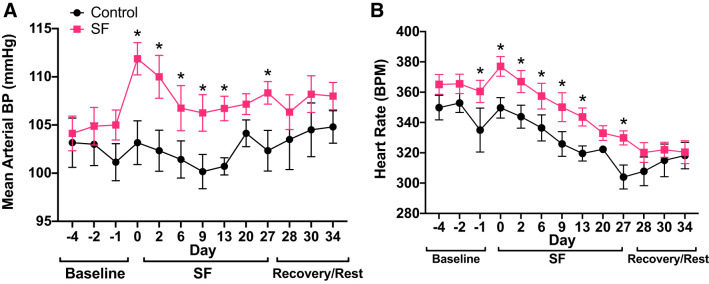
24 h mean arterial blood pressure (BP) in Control and SF rats. *A*: average of 24 h of mean arterial pressure (MAP) in intervention sleep fragmentation (SF) rats (pink) and control rats (black) during baseline and across intervention days. SF rats demonstrated significantly higher MAP when compared with controls at intervention *days 0–27* (with the exception of *day 20*). *B*: average of 24 h heart rate in intervention SF rats (pink) and control rats (black) during baseline and across intervention days. SF rats demonstrated significantly higher heart rate when compared with controls at intervention *days 0–27* (with the exception of *day 20*). bpm, beats/min. Data (means ± SE) were analyzed using mixed model analysis with repeated/fixed measures (time × SF/control groups) followed by post hoc multiple comparison tests with Sidak’s correction. *P < 0.05. *Days −4* to *13* include 7 control and 8 SF rats; *days 20* to *34* include 6 control and 6 SF rats.

At baseline, the 24 h MAP was similar between control and SF rats (post hoc Sidak *P* = 0.531, *P* = 0.404, and *P* = 0.126 at *days −4*, *−2,* and *−1*, respectively) and the SF was associated with increased MAP in intervention animals (group × time *F*_12, 120.86_ = 3.1, *P* = 0.001; Fig. 4*A*). On *day 0*, when the SF chamber was first activated, SF rats demonstrated a 9.4 ± 2.9 mmHg (post hoc Sidak, *P* = 0.006) increase in MAP, relative to their baseline values. Compared with controls, MAP was significantly higher in SF rats on *days 0*, *2*, *6*, *9*, *13*, and *27*. Sleep/h during the SF period was an independent predictor of MAP, and a 10 min reduction of sleep/h predicted an increase of MAP by 1.1 mmHg (*P* < 0.001). SF was also associated in an increase in heart rate in intervention rats compared with controls (group *F*_1, 117.49_ = 37.88, *P* < 0.001; time *F*_12, 17.75_ = 13.45, *P* < 0.001; Fig. 4*B*). Mirroring MAP, heart rate was significantly higher in SF rats versus controls on *days 0*, *2*, *6*, *9*, *13*, and *27*. Systolic and diastolic blood pressure values were also increased in intervention rats during the intervention period, compared with controls (Supplemental Table S8; https://doi.org/10.6084/m9.figshare.12323996). Systolic and diastolic blood pressure responses to SF followed the same pattern as MAP in both groups at *days 0–20*, but systolic and diastolic blood pressure were not increased versus controls at SF *day 27* (*P* = 0.071 and *P* = 0.224, respectively).

#### The Firmicutes-to-Bacteroidetes ratio and alpha diversity were decreased, and the relative abundance of Proteobacteria was increased in SF rats compared with controls during mid-SF.

Fecal microbial community structure was assessed using cultivation-independent sequencing of 16S rRNA gene amplicons. In total, 8,035,902 sequences were analyzed, with an average of 45,919 sequences per sample. A single failed sample (22 sequences total) was removed from the analysis. Sequence depth was rarefied to 25,502 sequences per sample for calculating alpha diversity indices. Unrarefied data were used for parametric differential abundance analyses performed using DESeq2. Data analyses were performed at different taxonomic levels, including phylum [e.g., calculating Firmicutes-to-Bacteroidetes (F:B) ratio], genus (alpha diversity and differential abundance), and at the operational taxonomic unit (OTU) level (differential abundance).

**Fig. 5.**
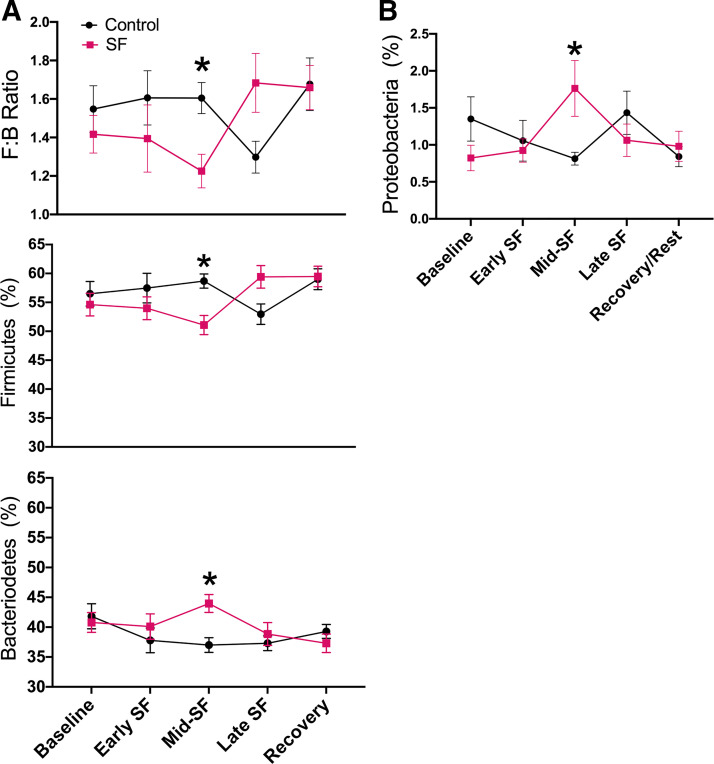
The composition of the gut microbiome changes in SF rats at the phylum level. *A*: in sleep fragmentation (SF) rats, the Firmicutes: Bacteroidetes (F:B) ratio decreases at mid-SF and returns to control levels at late SF. Data (means ± SE) were analyzed using mixed model analysis with repeated/fixed measures (time × SF/control groups) followed by post hoc multiple comparison tests with Sidak’s correction. Statistics for the F:B ratio were performed on log10 transformed data, while raw values are presented. *B*: the relative abundance of Proteobacteria was significantly elevated in SF animals versus controls at mid-SF. Data (means ± SE) were analyzed using mixed model analysis with repeated/fixed measures (time × SF/control groups) followed by post hoc multiple comparison tests with Sidak’s correction. Statistics for Proteobacteria performed on “−1/X” transformed data, while raw values are presented. Baseline includes 7 control and 8 SF rats (3 time points per animal); early SF includes 7 control and 8 SF rats (2 time points per animal); mid-SF includes 7 control and 8 SF rats (3 time points per animal); late SF includes 7 control and 6 SF rats (2 time points per animal); and recovery/rest includes 5 control and 5 SF rats (3 time points per animal). *P* < 0.05.

The F:B ratio has been used extensively as a proxy for broad shifts in microbial community structure from mammalian feces (56) and was similarly used here. We observed that while F:B ratios were not statistically different between treatment groups at baseline or early SF (*P* = 0.913), the F:B ratio was significantly lower for SF treatment rats at mid-SF (*P* = 0.012; Fig. 5*A*). In late SF and in recovery, the F:B ratio was not significantly different between treatment groups (*P* = 0.272 and *P* = 0.999, respectively). Rats exposed to SF had an average decrease of −0.06 units of the F:B ratio per day until mid-SF [95% CI (−0.06, −0.01), group × time *P* = 0.035], and an increase of 0.02 units per day from mid-SF through recovery [95% CI (0.01, 0.04), group × time *P* = 0.012] while the control rats did not. As food and body weight can affect the F:B ratio, we examined daily food intake, weight, and feeding efficiency in both groups, finding no differences between controls and SF rats (Table 1).

**Table 1.** 24 h food intake and weight in SF versus control rats

Day	Food Intake, g		*P*	Weight, g		*P*	Feeding Efficiency		*P*
	SF	Control		SF	Control		SF	Control	
*−4*	19.3 ± 0.8	17.7 ± 0.4	0.118	275.6 ± 9.1	285.1 ± 17.6	0.626			
*−2*	18.3 ± 1.0	19.0 ± 0.4	0.517	277.4 ± 9.1	286.7 ± 18.6	0.647			
*−1*	18.6 ± 1.2	18.3 ± 0.7	0.816	278.9 ± 9.1	290.7 ± 18.0	0.553			
*0*	18.5 ± 1.0	17.4 ± 0.9	0.440	276.0 ± 7.8	292.0 ± 16.6	0.379	−0.1 + 0.3	0.2 + 0.3	0.093
*2*	17.6 ± 0.6	17.9 ± 0.8	0.817	276.4 ± 7.8	296.0 ± 15.5	0.260	−0.1 + 0.3	0.3 + 0.3	0.172
*6*	19.8 ± 1.0	18.1 ± 0.7	0.228	282.3 ± 7.8	304.3 ± 15.4	0.206	0.3 + 0.3	0.9 + 0.3	0.172
*9*	17.5 ± 1.1	18.9 ± 1.1	0.409	285.3 ± 6.6	313.0 ± 13.8	0.081	0.5 + 0.3	1.2 + 0.3	0.137
*13*	20.8 ± 1.2	19.1 ± 0.9	0.302	297.0 ± 5.9	318.4 ± 13.7	0.593	1.0 + 0.3	1.6 + 0.3	0.397
*20*	19.7 ± 1.5	17.6 ± 0.8	0.226	316.5 ± 3.3	332.4 ± 9.9	0.182	1.8 + 0.3	2.4 + 0.3	0.213
*27*	19.7 ± 1.6	18.7 ± 1.6	0.675	330.2 ± 3.1	337.0 ± 12.3	0.602	2.4 + 0.3	3.2 + 0.4	0.199
*28*	19.2 ± 0.9	18.4 ± 0.9	0.581	335.2 ± 3.8	322.2 ± 13.5	0.340	2.7 + 0.3	3.1 + 0.4	0.565
*31*	21.0 ± 1.9	17.6 ± 0.8	0.146	337.4 ± 5.6	341.4 ± 13.4	0.789	2.8 + 0.4	3.5 + 0.4	0.364
*34*	20.4 ± 1.3	17.2 ± 0.7	0.063	345.2 ± 6.9	355.8 ± 16.4	0.539	3.2 + 0.4	3.9 + 0.4	0.176

Data are means ± SE and were analyzed by using mixed model analysis with repeated/fixed measures [time × sleep fragmentation (SF)/control groups] followed by post hoc multiple comparison tests with Sidak’s correction. Feeding efficiency calculated by daily weight gain (g) from baseline (averaged)/ daily food consumption (g). SF rats (*n* = 8–5), control rats (*n* = 7–5).

The relative abundance of sequences from the phylum Proteobacteria was also affected by SF (group × time *F*_4, 122_ = 2.58, *P* = 0.041; Fig. 5*B*). At baseline, the relative abundance of Proteobacteria was significantly higher in control animals relative to SF animals (*P* = 0.035; Fig. 5*B*). At mid-SF, Proteobacterial relative abundance was significantly higher in SF animals relative to control (*P* = 0.047), but this elevated abundance decreased during the late SF and recovery periods (*P* = 0.380 and *P* = 0.741, respectively). At mid-SF, differential abundance analysis demonstrated an increased relative abundance of sequences derived from *Escherichia-Shigella* in SF rats by 3.43 ± 0.71 compared with controls [log 2-fold change, false discovery rate-corrected (FDR) *P* < 0.001].

**Fig. 6.**
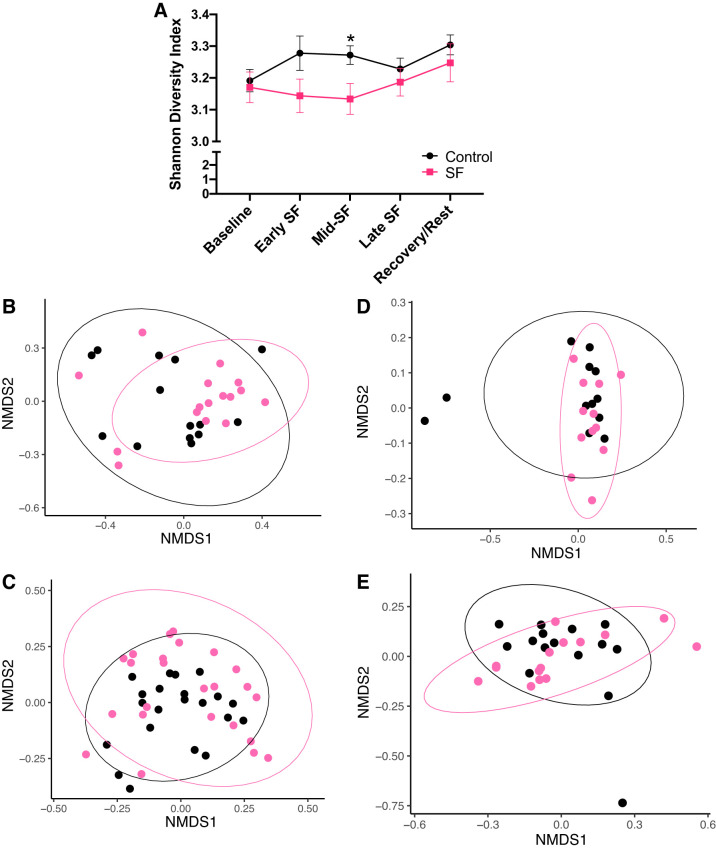
Alpha diversity of gut microbiota samples is decreased at midsleep fragmentation (SF) but overall community differences unchanged in SF animals. *A*: alpha diversity of gut microbiota samples is decreased at mid-SF in SF animals versus controls. Values represent means ± SE. Statistics performed on log10 transformed data. Values are expressed as raw numbers. *B–E*: nonmetric multidimensional scaling (NMDS) plots of fecal microbiome, performed at the taxonomic level of genus and based on Bray-Curtis dissimilarity. Overall gut microbial community structure was not significantly different between SF and control rats, as determined by analysis of similarity (ANOSIM) analysis, at *B*. Early SF (*R* = 0.06, *P* = 0.075), *C*: mid-SF (*R* = 0.01, *P* = 0.34), or D. Late SF (*R* = 0.03, *P* = 0.19). E. In recovery, there were small but significant differences in microbial community structure between control and SF rats (*R* = 0.10, *P* = 0.026). Analyses performed with relative abundance data filtered to 1% at the genus level. Baseline includes 7 control and 8 SF rats (3 time points per animal); early SF includes 7 control and 8 SF rats (2 time points per animal); md-SF includes 7 control and 8 SF rats (3 time points per animal); late SF includes 7 control and 6 SF rats (2 time points per animal); and recovery/rest includes 5 control and 5 SF rats (3 time points per animal). *P* < 0.05.

We further examined alpha and beta diversity metrics (genus level) to assess effects of SF on the total gut microbial community. Compared with controls, SF rats had lower alpha diversity (Shannon index) during early and mid-SF, and this was significant in mid-SF (*P* = 0.015; Fig. 6*A*). Subsequently, during late SF and recovery, microbial alpha diversity was not significantly different between groups (*P* = 0.997 and *P* = 0.365, respectively). Although there was a significant effect of microbial alpha diversity at mid-SF, microbial beta diversity analyses did not indicate effects of treatment on the observed microbial community structure at baseline (*R* = 0.04, *P* = 0.095) or during early, mid-, or late SF (Fig. 6, *B*–*D*; ANOSIM *R* < 0.07; *P* > 0.075). During the recovery/rest period, there was a small but significant difference between SF and control animals (ANOSIM *R* = 0.10, *P* = 0.026; Fig. 5*E*).

#### Several putative SCFA-producing bacteria were differentially abundant in SF rats compared with controls during mid-SF, late-SF, and recovery.

We hypothesized that the abundance of putative SCFA-producing bacteria in the gut microbiota would change during SF and therefore highlight these results in addition to untargeted analyses of all bacterial genera. In total 22 genus-level taxa were identified as significantly differently abundant at any stage (Table 2). At baseline, the relative abundance of *Bifidobacterium* was the only taxon that was significantly different between SF and control animals (FDR *P* = 0.023), but this difference was not maintained throughout SF or the recovery/rest period. The mid-SF, late SF, and recovery/rest periods were characterized by differences in the abundance of SCFA-producing bacteria in SF rats versus controls (Table 2). During early SF, no differences were found in the relative abundance of putative SCFA-producing bacteria. During mid-SF, however, SF rats had lower abundance in three genera containing putative butyrate producing bacteria, including *Eubacterium*, *Oscillospira,* and *Butyrivibrio,* compared with controls. During late SF, SF rats had higher relative abundance of *Dorea* and a lower relative abundance of *Parabacteroides*, which are both putative acetate-producing genera, than controls. During recovery and rest periods, SF rats had higher abundances of putative acetate (*Dorea, Anaerofustis*) and butyrate producers (*Subdoligranulum, Anaerofustis*), compared with controls (10). These changes were mirrored by similar differential abundance patterns of features at the sub-OTU level (Supplemental Table S4; https://doi.org/10.6084/m9.figshare.12149265).

**Table 2.** Effect of SF on gut microbiota differential abundance at genus level

Bacteria		Abundance, %		Effect (versus Control)	FDR *P*
Genus	Family	SF	Control		
*Baseline*					
*Bifidobacterium* ‡	Bifidobacteriaceae	0.019 ± 0.012	0.052 ± 0.025	−2.76 ± 0.75	0.023
*Early SF (days 0–3)*					
*Lachnospiraceae UCG 008*	Lachnospiraceae	0.380 ± 0.104	0.159 ± 0.033	1.54 ± 0.47	0.082
					
*Mid-SF (days 6–13)*					
*Eubacterium ruminantium group* §	Lachnospiraceae	0.003 ± 0.002	0.522 ± 0.213	−7.34 ± 1.12	<0.001
*Enterococcus*	Enterococcaceae	0.254 ± 0.224	0.007 ± 0.004	3.52 ± 0.67	<0.001
*Escherichia Shigella*	Enterobacteriales	0.692 ± 0.325	0.321 ± 0.200	3.43 ± 0.71	<0.001
*Butyrivibrio* †©	Lachnospiraceae	0.010 ± 0.004	0.058 ± 0.018	−2.94 ± 0.85	0.013
*A2*	Lachnospiraceae	0.230 ± 0.079	0.058 ± 0.018	2.45 ± 0.76	0.019
*Oscillospira* §	Ruminococcaceae	0.015 ± 0.003	0.028 ± 0.004	−0.94 ± 0.31	0.035
*Lachnospiraceae UCG 008*	Lachnospiraceae	0.398 ± 0.113	0.193 ± 0.027	1.18 ± 0.40	0.036
*Harryflintia*	Ruminococcaceae	0.031 ± 0.008	0.038 ± 0.010	−1.22 ± 0.42	0.036
*Late SF (days 20–27)*					
*Lachnospiraceae UCG 006*	Lachnospiraceae	0.191 ± 0.040	0.052 ± 0.012	1.86 ± 0.50	0.019
*Parabacteroides* ‡	Tannerellaceae	0.403 ± 0.142	1.236 ± 0.242	−1.70 ± 0.48	0.021
*Dorea*‡	Lachnospiraceae	0.288 ± 0.129	0.035 ± 0.014	2.89 ± 0.91	0.049
*Recovery/Rest (days 28–34)*					
*Ruminiclostridium 5*	Ruminococcaceae	0.588 ± 0.093	0.222 ± 0.027	1.48 ± 0.28	<0.001
*Subdoligranulum*§	Ruminococcaceae	0.006 ± 0.003	0.045 ± 0.009	−2.97 ± 0.74	0.002
*Harryflintia*‡	Ruminococcaceae	0.048 ± 0.019	0.031 ± 0.019	2.29 ± 0.57	0.002
*gut metagenome*	Mollicutes RF39 (order)	0.018 ± 0.007	0.152 ± 0.033	−3.24 ± 0.83	0.002
*Romboutsia*†	Peptostreptococcaceae	2.140 ± 0.261	1.037 ± 0.196	1.26 ± 0.32	0.002
*Ruminococcaceae UCG 005*	Ruminococcaceae	0.932 ± 0.180	2.685 ± 0.319	−1.55 ± 0.42	0.003
*Dorea*‡	Lachnospiraceae	0.268 ± 0.082	0.047 ± 0.017	2.73 ± 0.74	0.003
*Coprococcus 2*	Lachnospiraceae	0.041 ± 0.029	0.489 ± 0.206	−3.69 ± 1.08	0.007
*A2*	Lachnospiraceae	0.098 ± 0.042	0.018 ± 0.006	2.69 ± 0.80	0.007
*Faecalibacterium UBA 1819*§©®	Ruminococcaceae	0.260 ± 0.111	0.066 ± 0.028	2.24 ± 0.70	0.013
*Rothia*	Micrococcaceae	0.009 ± 0.003	0.034 ± 0.008	−1.93 ± 0.63	0.017
*Anaerofustis*†	Eubacteriaceae	0.021 ± 0.006	0.007 ± 0.002	1.87 ± 0.63	0.023
*Lachnospiraceae UCG 008*	Lachnospiraceae	0.128 ± 0.020	0.251 ± 0.034	−0.94 ± 0.33	0.030
*Ruminococcus 1*	Ruminococcaceae	0.795 ± 0.129	1.528 ± 0.164	−0.92 ± 0.33	0.031

Abundance values are expressed in relative percent taxa abundance of gut microbiota sample (means ± SE). Effect is log 2-fold change ± SE. SF, sleep fragmentation. FDR *P* is false discovery rate-corrected *P* value.

† Putative acetate-and butyrate-producing bacteria.

‡ Putative acetate-producing bacteria.

§ Putative butyrate-producing bacteria.

©Putative succinate-producing bacteria.

®Acetate-utilizing bacteria.

#### Total relative abundances of putative acetate- and succinate-producing bacteria were significant predictors of blood pressure.

In addition to SCFA-producing bacteria, we hypothesized succinate-producing bacteria would be associated with SF and MAP levels (67). Therefore, relative abundances of individual SCFA- and succinate-producing bacteria were stratified in groups based on expected metabolite end product (SCFAs [acetate, butyrate, and propionate] or succinate (Supplemental Table S2; https://doi.org/10.6084/m9.figshare.12149202), and relative abundances were compared. However, the relative abundance of grouped putative SCFA-producing bacteria were not significantly different versus controls at any time point (Supplemental Table S5; https://doi.org/10.6084/m9.figshare.12149289).

Relationships between the relative abundance of putative metabolite-producing taxa and MAP were examined through mixed-effect regression analysis. In both SF and control animals, grouped acetate-producing bacteria correlated with increased MAP and grouped succinate-producing bacteria correlated with decreased MAP (Supplemental Table S6; https://doi.org/10.6084/m9.figshare.12149328). Significant associations were observed for putative acetate-producing bacteria (*P* = 0.032) and succinate-producing bacteria (*P* = 0.006), but not butyrate- (*P* = 0.146), propionate- (*P* = 0.205), or mixed SCFA-producing bacteria (*P* = 0.418). In both SF and control rats, for each 1% increase in the relative abundance of acetate-producing bacteria, MAP increased by 1.30 mmHg [95% CI (0.11, 2.49), *P* = 0.032]. For each 1% increase in the relative abundance of succinate-producing bacteria, MAP decreased by 5.31 mmHg [95% CI (−9.13, −1.49), *P* = 0.006] and in SF and control rats together. *Faecalibacterium* sp. UBA 1819, a putative succinate-producing bacterium, was 2.24 ± 0.70 times more abundant during the recovery/rest period in SF rats versus control rats (FDR *P* = 0.013, Table 2). The relative abundance of grouped putative succinate-producing bacteria (Supplemental Table S5; https://doi.org/10.6084/m9.figshare.12149289) and levels of fecal succinate in metabolite analysis were elevated in SF rats versus controls (SF animals 0.57 ± 0.14 μmol/mg vs. control animals 0.28 ± 0.04 μmol/mg; *P* = 0.078). Although the effect was substantial, the observation was not significant at *P* = 0.05 level. Correlations were performed between the relative abundance levels of grouped putative-succinate producing bacteria and the untargeted metabolite analysis. In SF rats, the relative abundance of putative succinate-producing bacteria together was associated with fecal metabolite levels of propionate at recovery/rest *day 34* (r = 0.87, *P* = 0.054).

#### UDP-glucose was associated with higher blood pressure values and was significantly lower during recovery/rest in SF rats versus control rats.

**Fig. 7.**
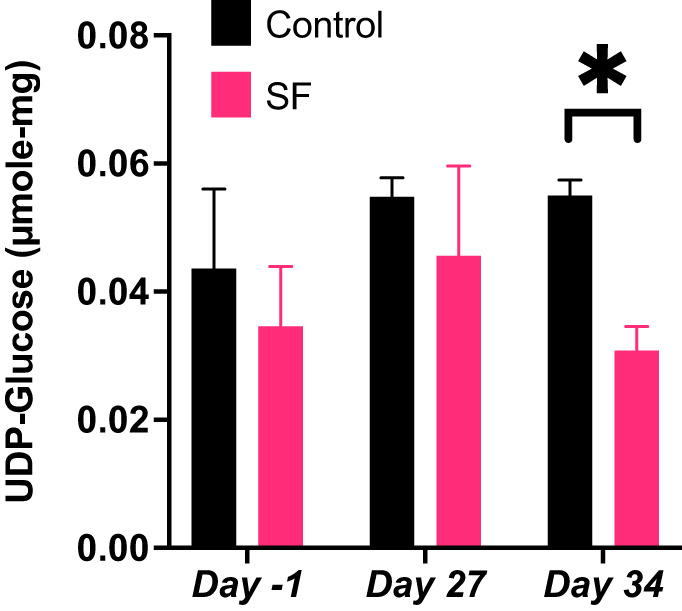
Fecal UDP-glucose is lower in SF rats versus controls during recovery/rest (*day 34*). Fecal UDP-glucose metabolite levels are not significantly different at baseline (*day −1*) or SF (*day 27*), but at recovery/rest (*day 34*), mean fecal UDP-glucose is lower in SF rats compared with control rats. Values represent means ± SE. Sample includes 5 control and 5 SF rats. *P < 0.05.

We sought to identify additional metabolites correlated with MAP using an untargeted metabolite analysis and mixed-effect regression models. This analysis revealed the fecal metabolite UDP-glucose was associated with a 4.70 mmHg increase in MAP [95% CI (0.29, 9.12), *P* = 0.037] when we controlled for time and treatment group (Table 3). Fecal UDP-glucose was not different between SF and control rats at baseline *day −1* or SF *day 27*, but during the recovery/rest period on *day 34* levels of UDP-glucose were significantly lower in SF rats (0.031 ± 0.004) compared with control rats (0.055 ± 0.002, *P* < 0.001, Fig. 7). Fecal SCFA metabolites (acetate, butyrate, and propionate) were not significantly positively or negatively associated with MAP (Supplemental Table S7; https://doi.org/10.6084/m9.figshare.2149358).1 Fecal metabolite levels of 3-hydroxyisovalerate, glutamine, and inosine were significant predictors of MAP (Table 3), and this finding was independent of study and treatment population.

**Table 3.** Mixed regression models of fecal metabolites and blood pressure outcomes

	Mean Arterial Pressure	
	Coefficient (95% CI)	*P*
2′-Deoxyinosine	−4.78 (−10.15, 0.58)	0.081
3-Hydroxyisovalerate	4.37 (1.26, 7.48)	0.006
3-Hydroxymandelate	−1.50 (−3.27, 0.27)	0.098
3-Phenylpropionate	0.78 (−0.12, 1.67)	0.085
Desaminotyrosine	−0.90 (−1.86, 0.05)	0.064
Glutamine	−0.29 (−0.50, −0.07)	0.010
Inosine	−2.98 (−5.92, −0.03)	0.048
UDP-glucose	4.70 (0.29, 9.12)	0.037

Models control for time and treatment group [time × sleep fragmentation (SF)/control groups]. Coefficients represent change in mean arterial pressure outcome per 0.1 unit increase in normalized fecal metabolite levels (μmol/mg). Samples includes 5 control and 5 SF rats.

## DISCUSSION

The present study is the first to demonstrate significant relationships among SF, MAP, and the gut microbiota. Our model system used rats with surgical telemetry implants to continuously record EEG/EMG and blood pressure and allow continuous measures of sleep and blood pressure. In our preclinical study, we focused on a possible mechanism linking disrupted sleep with blood pressure: alterations of metabolite-producing bacteria in the gut microbiota that may sustain or counteract MAP elevations in response to SF.

We report three novel findings: *1*) The degree of MAP increase is linked with the amount of sleep loss caused by SF; *2*) Mid-SF (*days 6, 9*, and *13*) changes in the gut microbiota of SF rats are characterized by reductions in alpha diversity, F:B ratios, and the relative abundance of putative butyrate-producing bacteria and by an increase in the relative abundance of Proteobacteria relative to control rats. Conversely, the relative abundance of putative acetate, butyrate, and succinate-producing bacteria increased during the recovery/rest (*days 28, 30,* and *34*) period; *3*) The relative abundance of putative acetate- and succinate-producing bacteria and several fecal metabolites in the gut microbiota was correlated with MAP outcomes.

Our SF intervention was effective in disrupting light-phase rat sleep, as evidenced by a significant decrease in sleep/h during the SF period. In previous studies attempting to cause SF or sleep restriction, investigators did not measure sleep by EEG/EMG monitoring (45, 64, 79). Using telemetry, we quantified sleep and included this variable in MAP and microbiota analyses. Sleep (min/h) during the 8 h SF period was a significant and independent predictor of 24 h MAP and a reduction of sleep by 10 min increased blood pressure by 1.1 mmHg. Although SF rats slept normally during the remaining hours of the day, we found that SF elicits elevated MAP even when recovery sleep occurs. SF is a prevalent and important health problem in humans that increases the risk for cardiovascular events by exerting a deleterious impact on autonomic function and ultimately blood pressure (18, 28, 36, 55, 80). Health problems and/or job-related schedules can restrict sleep to times when the circadian drive for wakefulness is high. Interestingly, MAP remained elevated in SF rats on *days 13* and *27,* even when sleep time during the bar on period was no different from controls. Although disrupted sleep has been associated with cardiovascular morbidity in several studies, these studies in humans are limited by the presence of unavoidable environmental population variance that complicate understanding of the mechanisms linking disrupted sleep with increased blood pressure and cardiovascular morbidity (55, 70).

Repeated-measures analysis of the gut microbiota at the taxonomic level of phylum provided insight into global changes in individual animals’ gut microbiota and gut dysbiosis. In animal models of hypertension, the F:B ratio has been shown to increase (2, 28) or decrease (31, 58) in comparison with control animals. In our study, the relative abundance of Firmicutes bacteria and the F:B ratio were lower in SF rats relative to controls at the same time when alpha diversity and butyrate-producing bacteria were also reduced. More severe models of SF (20 h/day) in a mouse model demonstrated an increase in the F:B after 5 days, but this study did not have an intermediate measure to determine if there was an initial decline in the F:B ratio similar to our study (13). Many putative SCFA-producing bacteria belong within the phylum Firmicutes, providing a possible explanation for the simultaneous rise in SCFA-producing bacteria and the relative abundance of Firmicutes bacteria at late SF (*days 20* and *27*). The relative abundance of Proteobacteria was increased at mid-SF in SF rats compared with controls, and this was driven by an increase in the relative abundance of *Escherichia-Shigella*. An increase in the relative abundance of Proteobacteria in the gut microbiome has been shown with other environmental insults such as high-fat diet or burn injury (21, 29), and an increased relative abundance of Proteobacteria has been associated with coronary artery disease and metabolic disorders in human studies (49, 81). These increases in proinflammatory Proteobacteria found in our research may have contributed to increased blood pressure through perpetuation of pathologic arterial and microvascular changes observed in other rodent models of SF (18). As mid-SF occurs when rodent sleep was still affected by SF and the increases in alpha diversity, the F:B ratio and Proteobacteria taxa at the genus and phylum level resolved by late SF when sleep normalized; these microbiome responses may be most closely associated with SF. Furthermore, an increase in Proteobacteria may be an important microbial biomarker and potential microbial target linked to SF that is worth further investigation in future research (82).

Changes in the gut microbiota are induced in rodent models of hypertension; most notably decreases in alpha diversity of gut microbiome samples and decreases of SCFA-producing bacteria have been shown (2, 28, 58, 73). In our study, we observed a decrease in alpha diversity in the gut microbiota samples of SF rats that corresponded with elevated MAP during mid-SF, similar to these prior studies. Also consistent with previous research (2, 28), we found that the relative abundance of putative butyrate-producing bacteria was reduced at mid-SF. This reduction in butyrate producers (seen in Table 2) occurred at the same time when both alpha diversity was decreased and MAP was elevated. Because the decrease in alpha diversity and relative abundance of putative butyrate-producing bacteria did not occur in the early SF period (*days 0–3*) when MAP was already elevated versus controls, the blood pressure changes evidenced during early SF do not appear to be a result of pathologic gut microbiome changes but a more direct effect of the SF intervention itself. Alternatively, effects of SF might initially alter the transcriptional response of the gut microbiota and be missed by DNA-based analyses. SF stimulates sympathetic nervous systemic activity and catecholamine release (38, 59), and the bacteria in the gut microbiota physiologically produce luminal norepinephrine (6). The increased 24 h MAP and heart rate during the intervention period suggest an increase in sympathetic nervous system activity related to SF. Interestingly, some pathogenic bacteria are also stimulated by norepinephrine (61), and the increased catecholamines potentially resulting from SF and sleep loss may be associated with the microbiota changes during mid-SF. Analysis of luminal and plasma norepinephrine could be quantified to determine if systemic SF-induced increases in norepinephrine are associated with luminal norepinephrine levels that could influence the gut microbiota community.

Specific putative acetate-, butyrate-, and succinate-producing bacteria were increased during late SF and recovery/rest (Table 2). The concept of gut microbial adaptation to exert protective effects on the host has been previously reported (19, 23), although in the present study it is unclear whether the gut microbiota changes had a measurable impact on MAP in the intervention animals. The increase in SCFA- and succinate-producing bacteria could be a response to SF-induced blood pressure elevations and previously demonstrated blood pressure-induced gut microbiota alterations (28, 58, 68). Projections from the paraventricular nucleus of the hypothalamus to the gastrointestinal tract provide neuroanatomical evidence of neural communication between the gut and cardiovascular regulatory centers in the brain (68). In our study, the relative percentage of putative succinate-producing bacteria was a strong predictor of MAP reduction, and this effect was independent of treatment group. Future research would benefit from evaluating if SF-induced increases in MAP could be abated by early supplementation of SCFA- or succinate-producing bacteria. Increased levels of microbiota-produced succinate has been shown to have metabolic effects such as improved glucose metabolism (23), and succinate exerts cardiovascular effects such as blood pressure reduction and reversed cardiovascular remodeling, possibly through its conversion to the SCFA propionate as demonstrated by the correlation between grouped succinate-producing bacteria and propionate metabolite levels (8, 63). Although corresponding increases in SCFA-metabolites were not found during recovery (Supplemental Table S5; https://doi.org/10.6084/m9.figshare.12149289), the increase in putative SCFA-bacteria is notable and requires future investigation. As SCFAs are both utilized and produced by bacteria in the gut (53), serial fecal metabolite profiling during SF may be necessary to further understand the dynamic relationship between the gut microbiota and fecal metabolites.

Metagenomic sequencing data suggest that even with individual differences in microbiota bacterial composition, metabolic pathways usually remain stable in the gut of healthy subjects (60). Additionally, recent research has shown that the microbial community phenotype, the functional result of metabolic pathways, is more suggestive of disease or heath status as compared with solely the presence or absence of bacteria (35). Therefore, in addition to characterization of the gut microbiome, we analyzed the fecal metabolome in a subset of rats before SF (baseline *day −1*) and on intervention *days 27* and *34* (7 days after SF was stopped). Higher fecal levels of SCFAs have been correlated with adiposity and hypertension in human studies (22, 39, 43) and high-salt diet in animal research (12). We observed increased succinate metabolite levels and increased grouped putative succinate-producing bacteria during recovery/rest in the SF rats, which corresponded to a twofold increase in *Faecalibacterium* sp. UBA 1819, a putative succinate-producing bacterium. Although group differences in the grouped succinate-producing bacteria and fecal succinate levels did not reach statistical significance between SF and control rats, the association between relative abundance levels of putative succinate-producing taxa and MAP was the strongest of any group observed (Supplemental Table S6; https://doi.org/10.6084/m9.figshare.12149328), and this was supported by metabolite analysis of succinate. Despite the relatively small number of animals and somewhat high variability in the fecal metabolite analysis, succinate and succinate-producing bacteria provided a strong signal that this metabolite may be linked to SF and blood pressure.

As untargeted NMR-based metabolomics analysis was performed, we discovered fecal metabolites that were significantly associated with MAP outcomes in addition to our hypothesized metabolite targets (SCFAs and succinate). Inosine was associated with decreased MAP in both SF and control rats. This compound is derived from adenosine and has been shown to have anti-inflammatory and cardioprotective properties in previous research (32, 52). The relationship between fecal levels of inosine and lower MAP related to vasoactive mechanisms are unknown at this point. Contrary to some prior studies (22, 43), we did not find fecal SCFA metabolites to be associated with MAP in our study. This may be due to the dynamic nature of microbial and metabolite interactions, or because samples were not collected at a time where sleep was altered in SF animals. There is conflicting evidence about whether changes in fecal levels of SCFAs, and possibly other bacterial metabolites, correlate with changes in cecal and plasma SCFA concentrations (9, 42), and plasma levels of SCFAs may be a better measure of metabolite effects on blood pressure (31). Including both systemic and fecal metabolite measures can be considered in future work to further understand relationships between the gut microbiome/metabolome and blood pressure.

UDP-glucose fecal metabolite levels were positively associated with increased MAP in both SF and control rats, and fecal UDP-glucose levels were significantly lower in SF rats versus control rats during the recovery/rest period (*day 34*). To our knowledge, no research has linked UDP-glucose and MAP outcomes, but UDP-glucose has been shown to produce porcine artery vasoconstriction through its action on P2Y_14_ receptors (1, 3). UDP-glucose’s vasoactive properties through cAMP-dependent signaling (3), and association with increased MAP suggests that this metabolite could be a promising target of future gut microbiota-linked hypertension research.

The present study contributes new knowledge about the pathophysiology of fragmented sleep and elevated MAP and provides a number of microbial and metabolite targets for further analysis. Although it appears that SF acts initially on blood pressure without gut microbial mediation, the gut microbiome is clearly impacted by extended SF. Thus, understanding the microbiota may be particularly important for identifying interventions targeting the gut microbiota that reduce cardiovascular morbidity in patients with sleep disorders. Augmenting the gut microbiota to increase or decrease levels of metabolites linked with reduced or elevated blood pressure levels, respectively, may prevent SF-induced blood pressure elevations when SF is unavoidable. The present preclinical investigation provides insights indicating that changes to the gut microbiota from SF are not unidirectional, and alterations in specific metabolite-producing bacterial populations are dependent on the “dose” of SF and blood pressure elevation. Early and late changes to the gut microbiota from SF-induced blood pressure elevation identify detrimental and protective gut bacterial responses to investigate for future therapies. Succinate-producing bacteria may be a novel target for preventing SF-induced blood pressure elevations before they occur. Limitations of this current work include the exploratory nature of the study and the small sample size. The research results, including the untargeted metabolomics, should be interpreted in light of these limitations. Nevertheless, this study lays a foundation and has identified important microbial and metabolite responses that can studied in future causational research.

## GRANTS

This work was supported by grants from the National Institutes of Health (NR014369 to A. M. Fink), International Society of Nurses in Genetics (to K. A. Maki), Sigma Theta Tau International (to K. A. Maki), Midwest Nursing Research Society to A. M. Fink), Janet Deatrick Research Award (A. M. Fink), and institutional funding from the University of Illinois at Chicago, Graduate College and College of Nursing (to K. A. Maki).

## DISCLAIMERS

The content is solely the responsibility of the authors and does not necessarily represent the official views of the National Institutes of Health.

## DISCLOSURES

No conflicts of interest, financial or otherwise, are declared by the authors.

## AUTHOR CONTRIBUTIONS

K.A.M. and A.M.F. conceived and designed research; K.A.M., M.W.-C., and D.S. performed experiments; K.A.M., L.A.B., and M.W.-C. analyzed data; K.A.M., L.A.B., M.W.C., L.E.R.-R., S.J.G., and A.M.F. interpreted results of experiments; K.A.M. prepared figures; K.A.M. drafted manuscript; K.A.M., L.A.B., M.W.C., M.W.-C., L.E.R.-R., S.J.G., and A.M.F. edited and revised manuscript; K.A.M., L.A.B., M.W.C., M.W.-C., D.S., L.E.R.-R., S.J.G., and A.M.F. approved final version of manuscript.
